# Manipulating the Level of Sensorimotor Stimulation during LI-rTMS Can Improve Visual Circuit Reorganisation in Adult Ephrin-A2A5^-/-^ Mice

**DOI:** 10.3390/ijms23052418

**Published:** 2022-02-22

**Authors:** Eugenia Z. Poh, Courtney Green, Luca Agostinelli, Marissa Penrose-Menz, Ann-Kathrin Karl, Alan R. Harvey, Jennifer Rodger

**Affiliations:** 1 School of Biological Sciences, The University of Western Australia, Crawley, WA 6009, Australia; e.poh@nin.knaw.nl (E.Z.P.); marissa.penrose@uwa.edu.au (M.P.-M.); karl_a1@ukw.de (A.-K.K.); 2 School of Human Sciences, The University of Western Australia, Crawley, WA 6009, Australia; 21956875@student.uwa.edu.au (C.G.); luca.agostinelli@research.uwa.edu.au (L.A.); alan.harvey@uwa.edu.au (A.R.H.); 3 Perron Institute for Neurological and Translational Research, 8 Verdun St, Nedlands, WA 6009, Australia; 4 Netherlands Institute for Neuroscience, Royal Netherlands Academy of Arts and Sciences, 1105 BA Amsterdam, The Netherlands; 5 Department of Neurology, University Hospital of Würzburg, Josef-Schneider-Strasse 11, 97080 Würzburg, Germany

**Keywords:** LI-rTMS, neuroplasticity, visual pathways, topography, visual activity, locomotion, brain state

## Abstract

Repetitive transcranial magnetic stimulation (rTMS) is a non-invasive brain stimulation technique that has the potential to treat a variety of neurologic and psychiatric disorders. The extent of rTMS-induced neuroplasticity may be dependent on a subject’s brain state at the time of stimulation. Chronic low intensity rTMS (LI-rTMS) has previously been shown to induce beneficial structural and functional reorganisation within the abnormal visual circuits of ephrin-A2A5^-/-^ mice in ambient lighting. Here, we administered chronic LI-rTMS in adult ephrin-A2A5^-/-^ mice either in a dark environment or concurrently with voluntary locomotion. One day after the last stimulation session, optokinetic responses were assessed and fluorescent tracers were injected to map corticotectal and geniculocortical projections. We found that LI-rTMS in either treatment condition refined the geniculocortical map. Corticotectal projections were improved in locomotion+LI-rTMS subjects, but not in dark + LI-rTMS and sham groups. Visuomotor behaviour was not improved in any condition. Our results suggest that the beneficial reorganisation of abnormal visual circuits by rTMS can be significantly influenced by simultaneous, ambient visual input and is enhanced by concomitant physical exercise. Furthermore, the observed pathway-specific effects suggest that regional molecular changes and/or the relative proximity of terminals to the induced electric fields influence the outcomes of LI-rTMS on abnormal circuitry.

## 1. Introduction

Non-invasive brain stimulation tools have the ability to improve cognition and performance in behavioural tasks in both healthy and patient populations. In particular, repetitive transcranial magnetic stimulation (rTMS) has demonstrated the therapeutic potential to treat a variety of neurologic and psychiatric conditions [1,2]. Because rTMS has the capacity to modulate neuronal activity, spontaneous and/or task-specific brain activities at the time of stimulation (i.e., brain state) [are likely to interact with the effects of rTMS. For example, studies in humans suggest that rTMS has a stronger impact on active versus inactive circuits: a single TMS pulse applied to an active muscle representation in motor cortex (e.g., active hand grasp) results in a much larger neural response, as measured with evoked potentials, compared to when the same pulse is applied to the cortex at rest (e.g., relaxed hand) [3]. While such interactions may contribute to the variability observed between, and within, subjects following rTMS-based therapies [4,5,6], any interplay between rTMS and brain state could potentially be harnessed for therapeutic purposes. Although rTMS is routinely applied to a patient at rest as an isolated treatment, there is increasing interest for magnetic stimulation to be used in synergy with conventional rehabilitative training [7,8,9,10,11,12].

Here, we have used a well-defined rodent visual system model to investigate how concurrent visual or locomotor input interacts with rTMS-induced plasticity in the visual pathway. In rodents, visual information captured by the retina is transferred to numerous centres in the brain, especially the contralateral superior colliculus (SC, optic tectum) of the midbrain, dorsolateral geniculate nucleus of the thalamus (dLGN) and primary visual cortex (V1) [13]. During development, guidance molecules are critical for the appropriate termination of developing axons. For example, bidirectional signalling between ephrin-A ligands, expressed on target brain regions, and EphA receptors, expressed on retinal and cortical axonal growth cones [14], are a key mechanism for accurate topographic mapping within the visual pathway [15,16]. Mice with homozygous null mutations of the ephrin-A2 and -A5 genes exhibit both normal and aberrant (ectopic) projections within retinotectal, geniculocortical and corticotectal projections [15,17,18,19,20]. We have previously shown that the visual pathway of transgenic mice lacking ephrin-A2 and -A5 ligands (ephrin-A2A5^-/-^) is susceptible to the effects of LI-rTMS [21,22]: fourteen consecutive days of LI-rTMS improves the topography of retinotectal and corticotectal projections and refines geniculocortical afferents in adult ephrin-A2A5^-/-^ mice [21,22]. Importantly, the repair of visual topography by LI-rTMS alone is incomplete, giving the model the capacity to detect both augmentation as well as diminution of the effects of rTMS when combined with additional interventions.

Previously, we showed that increasing the salience of visual information by training ephrin-A2A5^-/-^ mice to perform a visually engaging task during LI-rTMS prevented the beneficial reorganisation of the corticotectal map as compared to no-task subjects [23]. A possible explanation is that the combination of visually evoked activity, subthreshold stimulation induced by LI-rTMS [24] and the modulation of cortical activity by other factors including attention, motivation, and locomotion during the task [25,26], may have non-selectively strengthened both ‘normal’ and ‘ectopic’ projections within the corticotectal pathway [23].

Here, we aim to extend our current understanding of how endogenous brain activity interacts with the effects of LI-rTMS by using different types of environmental manipulation during each LI-rTMS session. We previously showed, using visual evoked potentials, that LI-rTMS applied to the visual cortex in the light has the opposite effect (excitation) compared to when it is applied in the dark (inhibition) [27]. Therefore, we delivered LI-rTMS to ephrin-A2A5^-/-^ mice in a dark environment to test the hypothesis that image-forming visual input is required for the beneficial effects of LI-rTMS on circuit refinement. In a separate cohort of mice, we examined the effect of concurrent locomotion on LI-rTMS effects. Although the lifetime benefits of physical exercise on the brain are well established in rodents and humans [28,29,30,31], evidence in rodents has demonstrated significant differences in activity in the primary visual cortex during periods of either quiescence or locomotion [26,32,33,34,35,36]. For example, locomotion (wheel running) indirectly increases the plasticity of adult primary visual cortex (V1) and can triple the rate of pyramidal neuron firing, to the point where voluntary running alone restores juvenile-like ocular dominance plasticity in mice [26,37]. A recent study suggests that similar cross-modal effects may also occur in humans, with physical exercise increasing cortical thickness in visual cortex [38]. Therefore, we delivered LI-rTMS during voluntary wheel running to test the hypothesis that locomotor activity would enhance the restorative effects of LI-rTMS on abnormal visual circuits. Understanding the impact of visual and physical activity on LI-rTMS effects provides unique insight into possible avenues to promote plasticity directly or indirectly in injured or dysfunctional brain regions.

## 2. Results

### 2.1. Corticotectal Projections

Thirty-eight out of 51 animals had at least one successful injection in V1 (Table 1; dark = 20/29 and locomotion = 18/22). The number of successful V1 injections was similar between stimulation groups (Table 1). Six animals were excluded due to fluorescent dyes impinging upon the underlying white matter tracts, and other injections were rejected because they were located in adjacent visual cortical areas, such as V2 (see Reference [39]).

**Figure 1:**
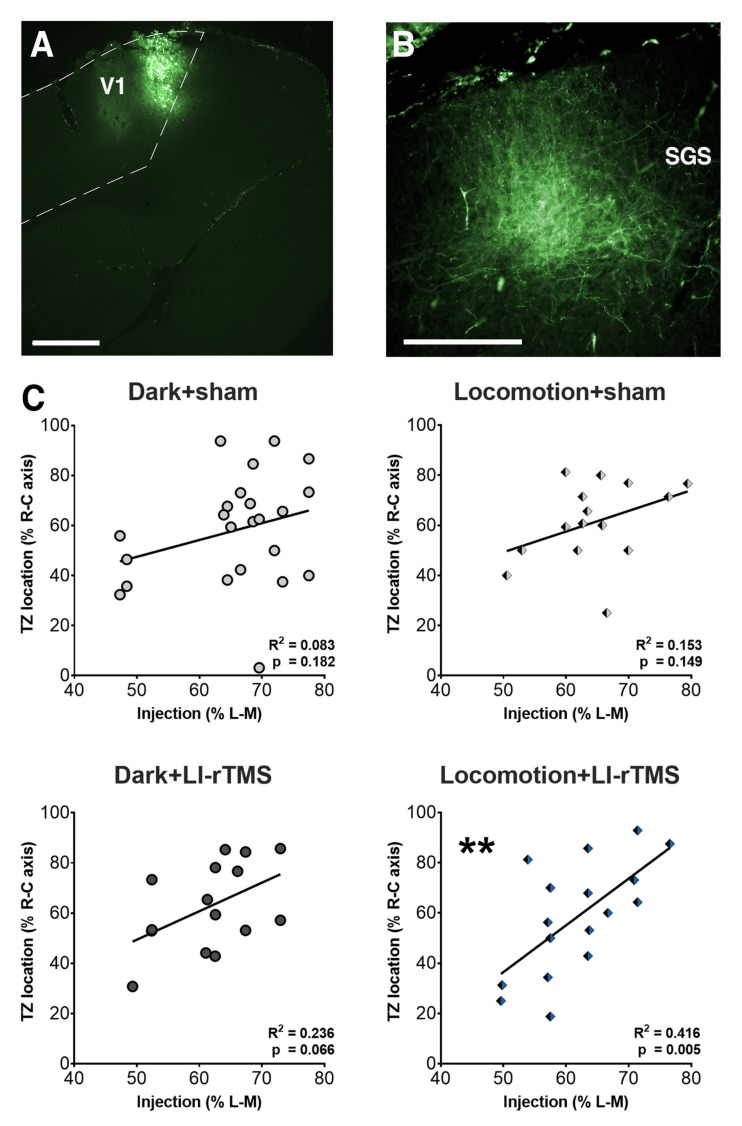
Linear regression analyses of injection and TZ location in ephrin-A2A5^-/-^ mice following chronic LI-rTMS. Sham or LI-rTMS was delivered to adult ephrin-A2A5^-/-^ mice for 14 consecutive days in a red-light room (dark) or with free access to a running wheel (locomotion). Example image of (**A**) injection site in V1 (scale bar = 250 µm) and (**B**) anterograde TZ labelling in the stratum griseum superficiale (SGS) in the SC (scale bar = 100 µm). (**C**) The location of fluorescent injections in V1 was expressed as a percentage of the lateromedial axis (L-M) of the hemisphere width, and TZ location was expressed as a percentage of the rostrocaudal axis (R-C) of the whole length of the SC. The coefficient of determination of the linear relationship between injection site in V1 and TZ location in the SC is presented. ** *p* < 0.01, linear regression analysis.

Anterogradely labelled terminal zones (TZs) were present in the ipsilateral SC, with the heaviest labelling in the stratum griseum superficiale [39] (SGS; Figure 1A,B). In some cases, we also observed multiple TZs in the SGS of SC following a single injection in V1 of ephrin-A2A5^-/-^ mice, which has been observed in previous studies [15,17,20]. It was found that chronic delivery of LI-rTMS in a dark environment (dark+LI-rTMS) or with concurrent locomotion (locomotion+LI-rTMS) did not significantly alter the proportion of TZs per V1 injection as compared to their sham counterparts (Fisher’s exact test; dark: *p* = 0.220, locomotion: *p* > 0.99).

To examine the potential reorganisation in the corticotectal circuit, we performed a linear regression analysis to determine whether there was a relationship between V1 injection location in the lateromedial axis and TZ location in the rostrocaudal axis. As expected, our results showed a very weak and nonsignificant linear relationship in sham-treated ephrin-A2A5^-/-^ mice in both groups (dark+sham: *F*_(1,21)_ = 1.906, *p* = 0.182; locomotion+sham: *F*_(1,13)_ = 2.354, *p* = 0.149; Figure 1C, top panels), consistent with previous results showing a lack of corticotectal topography in this strain [17,22,23]. Importantly, there was also a nonsignificant relationship in mice treated with online LI-rTMS in a dark environment (*F*_(1,13)_ = 4.026, *p* = 0.066; Figure 1C, bottom left). This finding is in contrast to our previous results showing a significant spatial relationship between the injection site and TZ location in ephrin-A2A5^-/-^ mice treated with online LI-rTMS under normal light conditions [23]. Finally, we observed a moderate and significant relationship between the injection site and TZ locations in LI-rTMS treated ephrin-A2A5^-/-^ mice that had free access to a running wheel during every stimulation session (*F*_(1,15)_ = 10.70, *p* = 0.005; Figure 1C, bottom right panel).

### 2.2. Geniculocortical Topography

The successful V1 injections also resulted in retrogradely labelled neurons in the ipsilateral dLGN. Thus, we also evaluated the dispersion of labelled dLGN neurons, measured using the convex-hull technique and expressed as a percentage of dLGN volume [22], area of the main cluster, and total number of labelled neurons. A two-way MANOVA (Pillai’s trace) showed a statistically significant effect of environment (i.e., locomotion and dark; *V* = 0.398, *F*_(3, 32)_ = 7.049, *p* < 0.001, observed power = 0.965), treatment group (i.e., sham and LI-rTMS; *V* = 0.518, *F*_(3, 32)_ = 11.472, *p* < 0.001, observed power = 0.998), and environment*treatment interaction (*V* = 0.398, *F*_(3, 32)_ = 7.049, *p* < 0.001, observed power = 0.852) on the combined labelled dLGN measures.

**Figure 2:**
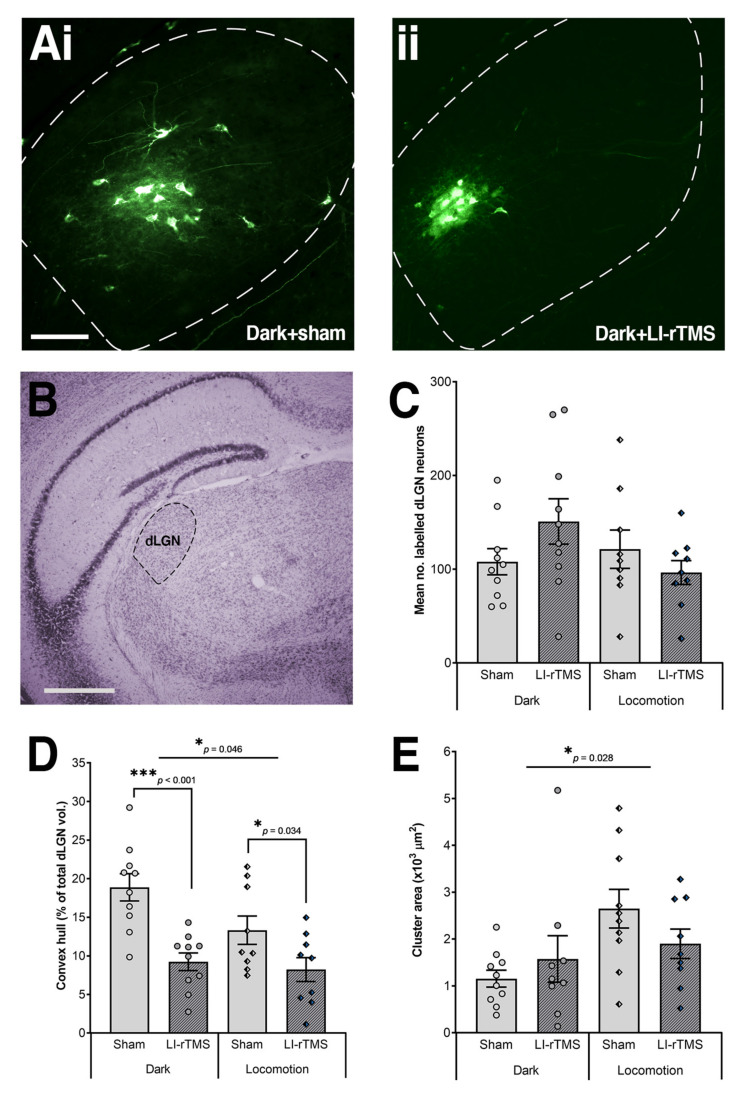
Geniculocortical neuron labelling of adult ephrin-A2A5^-/-^ mice following chronic LI-rTMS in a dark environment. Injections of fluorescent dye into V1 retrogradely labelled dLGN neurons. (**Ai**) Example image of disperse labelling in dark-sham-treated ephrin-A2A5^-/-^ mice, whereas (**Aii**) dark-LI-rTMS-treated mice exhibited less dispersion (scale bar = 100 µm). (**B**) Example image of dLGN identified in a cresyl-stained section (scale bar = 250 µm). (**C**) The average number of labelled dLGN neurons were not different between treatment (sham vs. LI-rTMS) and environment (dark vs. locomotion) groups. (**D**) The total dispersion volume of dLGN neurons was reduced by chronic online LI-rTMS. Interestingly, locomotion groups also had less overall dispersion volume as compared to subjects in the dark group. (**E**) Main cluster areas were not altered by LI-rTMS. However, locomotion groups had larger areas as compared to dark animals. Error bars represent SEM. Uppermost lines denote univariate analysis (* *p* < 0.05) and angled lines denote follow-up pairwise comparisons with Sidak correction (* *p* < 0.05, *** *p* < 0.001).

The univariate analyses revealed that environment had a significant main effect on cluster areas (*F*_(1, 34)_ = 5.240, *p* = 0.028, observed power = 0.604; Figure 2C) and convex-hull volumes (*F*_(1, 34)_ = 4.272, *p* = 0.046, observed power = 0.519; Figure 2D), but not the number of labelled dLGN neurons (*F*_(1, 34)_ = 1.218, *p* = 0.277, observed power = 0.189; Figure 2E). Follow-up pairwise comparisons with Sidak correction revealed that mice that had free access to running wheels had larger cluster areas (*p* = 0.028) and smaller convex-hull volumes (*p* = 0.046) compared to subjects given sham or LI-rTMS in a dark environment.

The treatment group had a significant main effect on convex-hull volumes (*F*_(1, 34)_ = 21.501, *p* < 0.001, observed power = 0.994) but not on any other measure (cluster area: *F*_(1, 34)_ = 0.078, *p* = 0.781, observed power = 0.059; number of labelled neurons: *F*_(1, 34)_ = 0.234, *p* = 0.631, observed power = 0.076). Follow-up pairwise comparisons with Sidak correction showed that online LI-rTMS, regardless of the environmental condition, reduced the abnormal dispersion of labelled dLGN neurons (vs sham, *p* < 0.001).

Despite the nonsignificant main effect of interaction between environment and treatment group for all dLGN measures (convex-hull volumes: *F*_(1, 34)_ = 2.042, *p* = 0.162, observed power = 0.284; cluster area: *F*_(1, 34)_ = 2.963, *p* = 0.094, observed power = 0.387; number of labelled neurons: *F*_(1, 34)_ = 3.307, *p* = 0.078, observed power = 0.424), we were interested in knowing whether chronic online LI-rTMS alters the abnormal dispersion of labelled dLGN neurons as compared to sham in their respective groups, a finding that has been observed in previous studies [22,23]. The pairwise comparisons with Sidak correction revealed a significant reduction in convex-hull volumes by chronic LI-rTMS in both dark (*p* < 0.001) and locomotion groups (*p* = 0.034). In sum, these findings suggest that chronic LI-rTMS reduces the large dispersion of labelled dLGN cells in ephrin-A2A5^-/-^ mice by selectively targeting the abnormally located dLGN neurons, and that image-forming visual input during LI-rTMS is not required for refining the geniculocortical map, nor does concurrent locomotion alter this effect.

### 2.3. Visuomotor Head Tracking

The optokinetic response is a measure of visuomotor function, integrating visual input from the retina to motor output in the SC [40]. The SC neurons are modulated by other afferent pathways, including corticotectal projections (i.e., V1 to SC) [41]. The ephrin-A2A5^-/-^ mice exhibited deficits in head tracking responses to moving visual stimuli in comparison to wildtype subjects (see References [21,42]). Here, chronic LI-rTMS delivered to ephrin-A2A5^-/-^ mice with reduced visual input did not improve visuomotor responses when compared to sham-treated animals (dark+LI-rTMS: 5.42 ± 0.65 tracks per minute (mean ± SEM); dark+sham: 4.47 ± 0.65; *U* = 49, *p* = 0.198). Furthermore, LI-rTMS with concurrent locomotion also did not increase the number of head tracking responses as compared to sham (locomotion+LI-rTMS: 2.56 ± 0.39; locomotion+sham: 2.12 ± 0.39; *U* = 40, *p* = 0.481). These results are in contrast to previous findings, where we have shown that LI-rTMS alone or in combination with a visually engaging task—both in normal light conditions—improves head tracking behaviour [21,23].

### 2.4. Concurrent LI-rTMS Does Not Alter Running Behaviour

**Figure 3:**
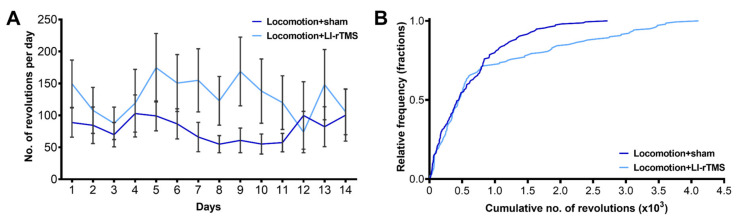
Online LI-rTMS did not alter the total distance ran during each session and over all sessions. Adult ephrin-A2A5^-/-^ mice were given free access to a running wheel during each treatment session. (**A**) Average revolutions on the running wheel per day were not different between sham or LI-rTMS treated subjects. Error bars represent SEM. (**B**) Cumulative number of running wheel revolutions over the 14 days.

We also assessed whether LI-rTMS altered voluntary running behaviour in ephrin-A2A5^-/-^ mice (Figure 3A). The cumulative distance ran by LI-rTMS-treated ephrin-A2A5^-/-^ mice was unaltered compared to sham (Mann-Whitney *U* = 9933, *p* = 0.245), and despite a rightward shift for LI-rTMS subjects (Figure 3B), the distributions were not significantly different between stimulation groups (Kolmogorov-Smirnov statistic = 1.329, *p* = 0.058). Thus, our current results suggest that online LI-rTMS does not alter this type of locomotor behaviour in ephrin-A2A5^-/-^ mice.

## 3. Discussion

The evidence from human [4,5,6] and preclinical studies [27,43,44,45] suggests that ongoing brain activity at the time of treatment has the capacity to modulate the outcomes of rTMS. Here, we show that chronic LI-rTMS in either the dark or locomotion treatment condition refined the geniculocortical map, while corticotectal projections were improved in locomotion+LI-rTMS subjects, but not in dark+LI-rTMS. Despite these various improvements to abnormal visual circuit reorganisation, the optokinetic, head tracking function was not improved in any condition. The observed differential effects on corticotectal and geniculocortical pathways suggest there may be unique regional molecular changes induced by LI-rTMS and/or brain state at the time of sensorimotor manipulation. In addition, our findings suggest that LI-rTMS does not alter voluntary running behaviour as compared to sham animals. This is similar to previous findings showing that chronic LI-rTMS does not alter general motor activity in ephrin-A2A5^-/-^ mice (see Reference [23]).

### 3.1. Corticotectal Pathway

Our study reveals that image-forming vision during LI-rTMS is necessary to drive beneficial map reorganisation in the corticotectal pathway, and that wheel running potentiates LI-rTMS effects in this pathway. A possible explanation for these findings is that LI-rTMS establishes a plastic environment which allows projections to be refined. The rTMS-induced changes to cortical physiology have been suggested to resemble those occurring during the critical or sensitive period of development [46] and may increase the capacity for plasticity in the mature brain [47,48,49]. For example, rTMS has been shown to increase the expression of brain-derived neurotrophic factor (BDNF) [21,22,50,51,52], disinhibit cortical circuits [53,54,55,56], and alter dendritic spine density in motor cortex [57]. Thus, the outcomes of rTMS-based therapies may modify and facilitate the removal of barriers to adult plasticity to create a more adaptive environment, in which endogenous brain activity can drive relevant and beneficial functional changes.

Combining LI-rTMS with wheel running would most likely enhance this plasticity because voluntary running has also been shown to reinstate processes similarly observed during the critical period of plasticity in the visual system [58,59,60], which includes the upregulation of BDNF expression [31]. An additional or alternative mechanism could be an increase in the visually evoked firing rate of neurons in the V1 as previously reported when mice transitioned from stationary to running [26]. Furthermore, previous research has shown that specific visual cortical circuits that are concurrently activated with locomotion can recover from monocular deprivation [37]. The findings suggest that rTMS may promote plasticity in neural circuits that are concurrently active with locomotion.

For ephrin-A2A5^-/-^ mice, image-forming visual input plays a key role because it likely identifies correctly located terminals amongst the disordered maps by eliciting the coincident firing of neighbouring retinal ganglion cells. In this way, the accurately located terminals may be reinforced through coincident activity with their neighbours, while aberrantly located terminals, which are active asynchronously with their neighbours, will be weakened or removed [41]. Therefore, removing the instructive role of visual information by stimulating ephrin-A2A5^-/-^ mice in the dark would not alter the ability of LI-rTMS to induce plasticity, but would prevent selective strengthening or weakening of terminals resulting in no improvement in corticotectal topography, as was observed in this study. Future experiments could investigate whether LI-rTMS during wheel running in the dark also results in a lack of reorganisation of the corticotectal map.

A key role of visual information in the form of coordinated retinal waves has been demonstrated previously in developing ephrin-A2A5^-/-^ mice [61]. When the ß2 subunit for the nicotinic acetylcholine receptor is knocked out in these mice (ephrin-A2A5ß2 triple knockout (TKO) mice), spontaneous retinal activity is present but does not display coordinated retinal waves [61,62,63,64]. Ephrin-A2A5ß2 TKO mice almost completely lack topographic order within the SC, indicating that ephrin-A-mediated guidance and patterned retinal activity are additive in establishing topography [61]. During development, V1 neurons project to the SC through the guidance of ephrin-As [15,18,61], but then align to retinotectal TZs by activity generated by retinal waves [20]. Although retinal waves are not present in adults, these new results suggest that coordinated visual activity in the form of image-forming visual input [22,23] is crucial for the refinement of the corticotectal map by LI-rTMS.

The interaction between LI-rTMS and activity in the corticotectal projection is complex. In a previous study, we attempted to enhance vision-related activity by training mice to engage in a visual learning task with LI-rTMS, but surprisingly, this combined intervention prevented the reorganisation of corticotectal projections [23]. A possible explanation is that the task likely involved other neural processes, such as the expectation of a reward, motivation, and active (versus voluntary) locomotion [25,26]. Together, these processes would have activated multiple cortical pathways, potentially confounding the instructive role of visual input in a highly active cortex. In support, studies have shown that the excitation of V1 neurons by low intensity TMS can sum with visually evoked activity to increase the sensitivity for detecting a weak visual stimulus, likely by increasing the response probability of sensory neurons [65]. Furthermore, BDNF secretion is linked to neuronal activity [66,67], and abnormally high levels of BDNF during development can induce the precocious onset and closure of the critical period [49]. Therefore, it is also possible that high BDNF levels in the SC induced by chronic LI-rTMS [22], combined with increases of BDNF due to high cortical activity from non-visual stimuli, may have not only overwhelmed or masked visuotopic information, but also limited the time window available for the reorganisation of ectopic terminations. Taken together, the experiments combining LI-rTMS with different visual environments suggest that too much or too little intrinsic activity within the visual cortex may interfere rather than assist the LI-rTMS-induced beneficial reorganisation of corticotectal visuotopic maps in ephrin-A2A5^-/-^ mice.

### 3.2. Geniculocortical Pathway

In contrast with the corticotectal projection, the geniculocortical map in ephrin-A2A5^-/-^ mice is consistently refined by LI-rTMS in all of our studies to date, regardless of concurrent visual input. A possible explanation is that differences in the time-course of BDNF expression following acute and chronic stimulation may underlie the pathway-specific effects of LI-rTMS. It was shown that BDNF levels within V1, the main cortical target of dLGN neurons, were transiently upregulated 2–24 h after the first stimulation session in ephrin-A2A5^-/-^ mice, but returned to baseline after 14 days of stimulation [22]. In contrast, BDNF within the SC, which contains the terminals of corticotectal projections, was upregulated both transiently and at a chronic timepoint [22]. The temporal aspects of BDNF signalling can affect downstream signalling cascades [68], for example through interactions with its receptors tyrosine receptor kinase B (TrkB) and p75 neurotrophin receptor (p75^NTR^) [68]. It is therefore possible that the region-specific upregulation of BDNF at specific times during LI-rTMS treatment, and resulting interactions with TrkB and/or p75^NTR^, may underlie some of the pathway specific effects of LI-rTMS observed in the present and previous studies. BDNF signalling may also contribute to differences in refinement between the most normal and abnormal terminations: the cluster area represents the location of neurons that have the most normal terminations, whereas the convex-hull measurement includes neurons with very abnormal geniculocortical projections. Therefore, by reducing the convex-hull area, locomotion+LI-rTMS may have preferentially refined the terminals of the most abnormally projecting dLGN neurons, while at the same time promoting branching of the more accurate terminations to increase the cluster area. We previously speculated that while BDNF generally strengthens strong and active connections through TrkB signalling, the inaccurate terminations in ephrin-A2A5^-/-^ mice might be more susceptible to removal due to neuronal nitric oxide synthase and p75^NTR^-mediated signalling [21]. These contrasting activity-dependent mechanisms could be further enhanced by heightened cortical excitability during locomotion, contributing to opposing patterns of refinement in accurate and inaccurate terminations in the geniculocortical projection.

The extent of reorganisation may also depend on the relative distance of the coil to the different neuronal compartments, which determines the intensity of the induced electric field. Because the effects of rTMS are highest at the surface of the cortex and the SC [69], the strongest induced electric field is likely received by the dendrites, cell bodies, and terminals of corticotectal projections, but only the axon terminals of the geniculocortical projections. Recent computational models of high-intensity TMS pulses have suggested that the axon terminals are more likely to be modulated than the neuronal cell bodies or dendritic compartments [70]. In contrast, earlier computational studies have suggested that cell bodies are more likely to be modulated by rTMS [71,72]. It is also unclear what influence stimulating the whole cell vs. only one compartment might have on neural circuit plasticity. Thus, further investigation is required to determine the biological relevance of these compartment-specific effects on the silencing and/or pruning of abnormally located compartments within V1, but not SC.

### 3.3. Head Tracking Behaviour

An important difference between our study and previous experiments is that chronic LI-rTMS delivered to freely moving ephrin-A2A5^-/-^ mice in the dark, or during locomotion in the light, did not improve visuomotor headtracking behaviour, even though topographic order had been improved in at least one relay of the visual pathway. It is perhaps not surprising that the absence of light prevented the restoration of visual function, given the importance of image forming vision in guiding functionally relevant plasticity (discussed above in Section 4.1). Consistent with this, completing a visual task concurrently with LI-rTMS does result in improved head tracking [23]; however, the finding that locomotion prevented the restoration of visual function that would normally be induced by LI-rTMS in normal light was unexpected. Given the significance of relevant visual input during LI-rTMS, and the lack of reorganisation with wheel running, perhaps the lack of optic flow during this running activity (animals remain stable relative to their environment) resulted in a mismatch between expected and actual visual input, compromising plasticity in visuomotor function. The implication is that increasing activity via visual-specific tasks results in functional restoration, while increasing activity through a non-visual modality is not effective and may even be disruptive. Our findings suggest that the modality used to increase activity in different parts of the CNS is highly relevant, and support data from physiotherapy approaches in patients, where training protocols need to be task-specific for a particular type of rehabilitation therapy in order to obtain optimal functional outcomes [73,74].

Within the visual system, the restoration of head-tracking behaviour may not require accurate topographic organisation throughout the visual pathway [21,23]. In previous experiments, improvements in head tracking were associated with improved retinogeniculate and retinocollicular topography [21], and with improved geniculocortical topography alone [23], suggesting redundancy in the control of head tracking behaviour. Here we find no improvements in head tracking behaviour despite the reorganisation of the corticotectal and/or geniculocortical pathway. In rodents, the optokinetic reflex serves to stabilise the retinal image mediated by the SC [40], and involves the integration of retinal and cortical inputs with motor output to control head movement [13,75]. Therefore, it is possible that accurate topography of other retinofugal projections, such as those projecting to pretectal structures [13], is a key requirement for headtracking behaviour.

## 4. Materials and Methods

### 4.1. Animals

All experiments were performed in accordance with the National Health and Medical Research guidelines and approved by The University of Western Australia Animal Ethics Committee (AEC 100/1639). The ephrin-A2A5^-/-^ mouse line was a generous gift from Dr David Feldheim and was backcrossed onto C57BL/6 mice for >20 generations. The mice were bred from heterozygous parents and genotyped at weaning [15] (*n* = 51). All animals were of adult age during rTMS delivery (M *=* 20 weeks old), of either sex (counterbalanced between treatment groups), and housed in 12 h light/dark cycle with food and water provided ad libitum. Following the coil attachment surgeries, the grids were removed from cages to prevent the attachments from being trapped [23]. Hydrogel (Necta H2O, Able Scientific, Canning, Australia) was given as a water substitute for the remainder of the experiment.

### 4.2. Online LI-rTMS of Freely Moving Mice

#### 4.2.1. Coil Support Surgeries

**Figure 4:**
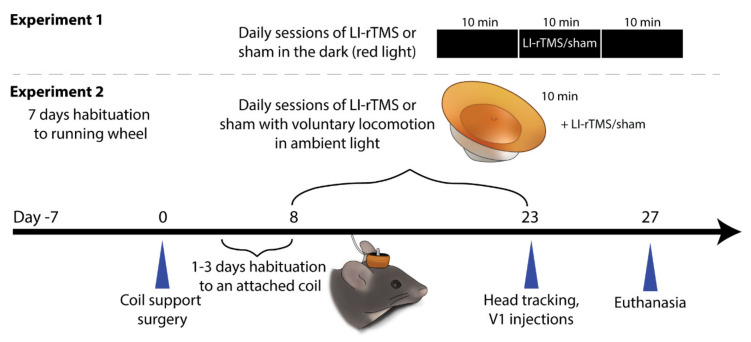
Experimental timeline of the current study. Adult ephrin-A2A5^-/-^ mice were divided into two environmental conditions. In Experiment 1, the mice were able to freely move in a dark environment and in Experiment 2, they were given free access to a running wheel in ambient light. In both experiments, the mice received either LI-rTMS or sham for 14 daily 10 min sessions.

Matched numbers of male and female ephrin-A2A5^-/-^ mice were allocated into each treatment group. As described previously [23], the mice were deeply anaesthetised (ketamine; 75 mg/kg; and medetomidine; 1 mg/kg i.p.; Troy Laboratories, Glendenning, Australia) and a coil support was attached above the lambdoid suture of the skull using cyanoacrylate (UHU, Bühl, Germany) and dental cement (Paladur, Heraeus Kulzer, Hanau, Germany). Following the attachment, the wound was sutured shut and lignocaine applied liberally on the surface of the surgical site. Anaesthetic reversal (atipamezole; 10 mg/kg s.c.; Troy Laboratories, Glendenning, Australia) and buprenorphine (0.05 mg/kg s.c.) were injected and the mice were monitored regularly for the first 6 h post-surgery. The coil support allowed the experimenter to deliver rTMS at acute and chronic (>1 week) timepoints in awake and freely moving mice, reducing potentially confounding factors related to restraining or anaesthetising mice during stimulation (Figure 4).

The custom-made coil comprised of 300 windings of copper wire (0.125 mm in diameter) with an inner and outer diameter of 6 and 8 mm, respectively [21]. The coil was connected to an electromagnetic pulse generator (e-cell^TM^) programmed to deliver LI-rTMS pulses using a biomimetic high frequency stimulation protocol (BHFS) for 10 min that was based on endogenous cellular activity associated with exercise (PCT/AU2007/000454; Global Energy Medicine). This involved 59.9-ms trains of 20 pulses (pulse width = 275 µs) at three different frequencies as follows: 1 min at 6.71 Hz, 8 min at 10.1 Hz, and 1 min at 6.26 Hz [21]. Three coils were used interchangeably for these experiments, with an average maximum (pulse width = 275 µs). Three coils were used interchangeably for these experiments, with an average maximum field intensity of 18.92 ± 1.49 mT (*M* ± *SD*) at the base of the coil. Sham-treated animals had a coil attached but the pulse generator was switched off.

#### 4.2.2. Red-Light (Dark) Environment

On the fifth to seventh day post-surgery, a sham coil was attached to the mice to habituate them to the coils. The stimulation procedure commenced on the eighth day post-surgery: the mice were moved to a different room under red-light illumination and habituated for at least 10 min before sham or real LI-rTMS treatment in their home cages. Mouse retinae lack long wavelength sensitive opsins [76]. Thus, a red-light environment is essentially perceived as a dark environment, and any responses are non-image forming [76]. The mice remained in the red-light room for another 10 min after each treatment session before being returned to the room with normal day-night light conditions. LI-rTMS or sham were delivered for 14 consecutive days.

#### 4.2.3. Concurrent Locomotion

This intervention was carried out under normal illumination (ambient lighting). Naïve mice (pre-surgery) were placed into a plastic box (W × L × H: 28.5 × 40 × 27 cm) with a wireless running wheel (MedAssociates, Inc.: ENV-044) positioned in the centre for 30 min per day for seven consecutive days. They were allowed to freely move around and explore the environment. Running data were collected with the accompanying Wheel Manager Software.

On the fifth to seventh day post-surgery, a sham coil was attached to the mice to habituate them to the coils. The LI-rTMS coils were placed above the mouse head by situating the coil on the support with an alligator clip to hold it in place. The mice were carefully placed into the box containing the running wheel. The LI-rTMS device was either turned on (LI-rTMS) or kept off (sham/control). Once the 10 min were completed, the alligator clip was unclipped, and the coil removed from the support. The mice received LI-rTMS or sham combined with locomotion for 10 min daily for 14 days. The number of revolutions completed during each stimulation session was recorded for each mouse and converted to distance in metres. The data were assessed as relative frequency (fractions) of the number of revolutions ran over the total revolutions ran over the 14 days.

### 4.3. Cortical Injections

After two weeks of daily LI-rTMS or sham, cortical injections of fluorescent tracers were performed to map the topography of corticotectal and geniculocortical projections. The mice were deeply anaesthetised (ketamine; 75 mg/kg; and medetomidine; 1 mg/kg i.p.; Troy Laboratories, Glendenning, Australia), placed in a stereotaxic frame, and the coil supports removed. Small pieces of skull and dura were removed to expose the left V1. Injection sites were determined visually using a landmark branch of the middle cerebral artery and confirmed using stereotaxic coordinates [77]. A Nanoliter 2010 (World Precision Instruments, Sarasota, FL, USA) with a micropipette was used to pressure inject two 300 nL (6 × 50nL) injections of biotinylated dextran amine (BDA; 10,000 MW; Thermo Fisher Scientific, Waltham, MA, USA) with Alexa Fluor 488 (green) and Alexa Fluor 555 (red) into lateral and medial V1, respectively, 400 µm from the surface of V1 targeting layer 5 pyramidal neurons projecting to the superficial grey layer of the SC. The injection sites were primarily within the monocular zone of V1; however, some injections were more lateral and hence likely to be within the binocular field [13]. Differences in corticotectal terminal zone (TZ) labelling based on monocular or binocular zone V1 injections have not been reported previously, and in the present study did not show any differences between red or green labelled TZs within the superficial grey layer of the SC, consistent with a previous rodent study [39]. Preliminary analyses showed that total injection volumes were not significantly different between colours (488 vs. 555) or stimulation groups (data not shown, all *p* > 0.05).

### 4.4. Anatomical Tracing Analyses

Four days after the cortical injections, the animals were terminally anaesthetised using sodium pentobarbitone (0.1 mL, i.p.; Lethabarb, Virbac, Australia). The mice were transcardially perfused with saline (0.9% NaCl *w*/*v*) and paraformaldehyde (4% in phosphate buffer, *w*/*v*). Whole brains were collected and postfixed in paraformaldehyde for 24 h, cryoprotected in sucrose solution (30% in PBS *w*/*v*), and cryosectioned coronally (40 µm) in three series. One series was imaged (Nikon DS-Qi2 camera, software: NIS-Elements Basic Research) and analysed using a Nikon e-800 fluorescence microscope to visualise anterogradely labelled TZs in the superficial grey layer of the SC, retrogradely labelled dLGN neurons, and fluorescent injection sites in V1 layer 5. The brain regions were confirmed using adjacent Nissl-stained sections in the second series. In cases where the fluorescent labelling of TZ was ambiguous (e.g., due to section damage), adjacent sections from the third series were examined with fluorescence microscopy.

#### 4.4.1. Topography of Corticotectal Projections

The topography of the visual field is maintained throughout the visual system [13]. We assessed topographic accuracy across the horizontal axis, which is mapped from the lateromedial axis of V1 across the rostrocaudal axis in the superficial grey layer of the SC [17,20,41]. During development, ephrin-A-EphA interactions map the horizontal (naso-temporal) visual axis, and the removal of the ephrin-A2 and -A5 ligands has been shown to disrupt this axis the most [78]. The laterality of the injection site in V1 was determined by measuring the distance from the centre-point of the injection to the medial cortical edge and expressed as a percentage of total cortical hemisphere width in order to normalise measures to brain size. The rostrocaudal TZ location was measured as the distance (µm) from the central section of the TZ rostrocaudal span to the caudal end of the SC, expressed as a percentage of total SC span (number of sections multiplied by section thickness). Multiple TZs were scored if separated by at least one section within a single series (i.e., 120 µm) or visually distinct from an adjacent TZ. Because not all injections resulted in ectopic TZs, each injection was analysed separately.

#### 4.4.2. Dispersion of Geniculocortical Neurons

The ipsilateral dLGN was analysed to determine whether delivering LI-rTMS in the dark reduced the scatter of retrogradely labelled neurons in this nucleus. In wildtype mice, an injection into V1 retrogradely labels a focal, topographically appropriate cluster of cells in the dLGN [17,22]. However, in ephrin-A2A5^-/-^ mice, the clusters are present but with a smaller number of cells situated outside the focal region [17,22]. Therefore, we assessed the dispersion of labelled dLGN cells in two different ways: convex-hull and main cluster area.

Convex-hull: The total area of the boundary containing the outermost labelled cells in each section of one series and multiplying by inter-section distance (120 µm) to obtain convex-hull volume (µm^3^) [22]. Convex-hull volumes were normalised to total dLGN volume to account for variation in dLGN size between animals. The total dLGN volume (µm^3^) was measured from images of Nissl-stained sections.

Main cluster: The labelled cluster size (µm^2^) in the dLGN was averaged over three sections per animal, selected from the middle span of dLGN sections with labelled cells to avoid sparse and uneven labelling toward rostral and caudal limits [22]. The number of labelled dLGN cells was also counted for each section in one series and multiplied by the number of series, in accordance with stereological principles.

Similar to previous reports [22,23], we confirm that there was no statistical difference between medial and lateral injections in the number of labelled dLGN neurons (data not shown, *p* > 0.05). Therefore, all dLGN measures were averaged within the mice.

### 4.5. Visuomotor Head Tracking

The visuomotor head-tracking was assessed by examining head-tracking behaviour in response to moving gratings on the day after the final LI-rTMS or sham stimulation [79,80]. The mice were placed on a stationary central pedestal within a motorised optokinetic drum consisting of rotating black and white vertical gratings (1 Hz: 0.13 cpd). The light intensity was maintained at 900–1100 lux throughout testing. The mice completed four trials of 120 s each, alternating the grating rotation between clockwise and anticlockwise, with 30 s rest between trials. The tests were video recorded, and the number of head-tracks per minute (>1 sec) was averaged across each session [21,42].

### 4.6. Statistical Analysis

The raw data were processed using Microsoft Excel and statistical analyses completed using SPSS (version 24.00, IBM) and Prism 9 (GraphPad Software).

For the anatomical tracing analyses, group differences in the proportion of ectopic TZs per injection were assessed by a two-tailed Fisher’s exact test within each brain state group (i.e., dark or locomotion). Linear regression was used to assess corticotectal topographical accuracy, with each injection site location (% in the lateromedial axis) considered independent and plotted against TZ locations (% in the rostrocaudal axis). A two-way multivariate analysis of variance (MANOVA) was used to examine the effect of brain state (dark, locomotion) and treatment (sham, LI-rTMS) on measures of retrogradely labelled dLGN neurons. Due to the violations of parametric assumptions, the number of head tracks per minute was assessed using Mann-Whitney *U* tests between LI-rTMS and sham. The locomotion was assessed as cumulative revolutions completed over the 14 days of stimulation. Since the data did not meet the assumptions of parametric statistical tests, we used two-sample Kolmogorov–Smirnov tests to compare overall distributions and compared medians using Mann–Whitney *U* tests between LI-rTMS and sham. The statistical significance was set at the alpha value of 0.5.

## 5. Conclusions and Future Directions

There is increasing interest for magnetic stimulation to be used as an adjuvant to conventional rehabilitative training [7,8,9,10,11,12]. The findings of the present study, in addition to previous results [21,22,23], suggest that intrinsic brain activity at the time of stimulation can modulate chronic LI-rTMS-induced structural neuroplasticity in abnormal adult visual networks, but the impact on visual function remains less clear. Importantly, interventions that interfere with vision during rTMS treatment may prevent beneficial functional outcomes, even while increasing structural neuroplasticity. Future studies are needed to elucidate the mechanisms underlying rTMS-induced neuroplasticity, for example, through changes in cortical excitation-inhibition and/or the recruitment of neurotrophic signals in specific visual pathways. It would be interesting in future studies to consider the application of low frequency stimulation paradigms in models where it is desirable to decrease cortical excitability. For example, 1 Hz stimulation has been shown to decrease cortical excitability in human and animal models [81,82], and alter dendritic morphology in mouse hippocampal slices [83]. Combining different tasks and stimulation paradigms may allow the selective enhancement of specific plasticity processes, leading to optimal repair. Finally, extending this research to other neuromodulation techniques that are commonly applied online such as transcranial direct current stimulation (tDCS) and transcranial alternating current stimulation (tACS), which have also been shown to modulate visual responses [84], would offer further insight into mechanisms and may lead to more options for translation.

## Figures and Tables

**Table 1:** Number of successful layer 5 V1 injections and labelled corticotectal TZs in ephrin-A2A5^-/-^ mice. Two colours refer to the successful labelling of TZs following medial Alexa Fluor 555 and lateral Alexa Fluor 488 injections.

		Dark		Locomotion	
		Sham	LI-rTMS	Sham	LI-rTMS
No. of successful injections per subject	One colour	8	8	6	6
	Two colours	2	2	3	3
Total no. of injections		12	12	12	12
No. of TZs per successful injection	=1	4	8	9	8
	>1	8 (67%)	4 (33%)	3 (25%)	4 (33%)

## Data Availability

Data available upon request from lead and corresponding authors.
